# Comprehensive three-dimensional positional and morphological assessment of the temporomandibular joint in skeletal Class II patients with mandibular retrognathism in different vertical skeletal patterns

**DOI:** 10.1186/s12903-022-02174-6

**Published:** 2022-04-28

**Authors:** Saba Ahmed Al-hadad, Enas Senan ALyafrusee, Abbas Ahmed Abdulqader, Waseem Saleh Al-gumaei, Rana A. A. M. AL-Mohana, Liling Ren

**Affiliations:** 1 Department of Orthodontics, School of Stomatology, Lanzhou University, Lanzhou, China; 2 Department of Orthodontics, West China School of Stomatology, Sichuan University, Chengdu, Sichuan China; 3 Department of Orthodontics and Dentofacial Orthopedics, Faculty of Dentistry, Ibb University, Ibb, Republic of Yemen; 4 Department of Orthodontics, Affiliated Stomatological Hospital of Jiamusi University, Jiamusi, China

**Keywords:** Temporomandibular joint, Retrognathism, Class II, Three-dimensional, Vertical skeletal patterns, CBCT

## Abstract

**Background:**

Only a few studies have used 3D cone-beam computed tomography (CBCT) analysis to evaluate the positional and morphological characteristics of the temporomandibular joint (TMJ) in adults with skeletal Class II. No studies have focused on the case of skeletal Class II with mandibular retrognathism in different vertical skeletal patterns. As a result, this study aimed to evaluate and compare the position and morphology of TMJ in adults with skeletal Class II with mandibular retrognathism in different vertical skeletal patterns to the position and morphology of TMJ in the normal Chinese adult population in three dimensions.

**Methods:**

This retrospective study analyzed CBCT images of 80 adult patients. Subjects with skeletal Class II with a normal sagittal position of the maxilla and mandibular retrognathism were classified according to the mandibular angle and facial height ratio into three groups of 20 subjects each: hypodivergent, normodivergent, and hyperdivergent groups, as well as a control group of 20 subjects. The following 3D measurements of TMJ were evaluated: (1) position, parameters, and inclination of the mandibular fossa; (2) position, parameters, and inclination of the mandibular condyle; (3) condyle centralization in their respective mandibular fossae; (4) anterior, posterior, superior, and medial joint spaces; and (5) 3D volumetric measurements of the TMJ spaces. Measurements were statistically analyzed by one-way ANOVA test, followed by Tukey’s post hoc test.

**Results:**

Significant differences were found in the hyperdivergent and hypodivergent groups compared with the normal group in the vertical and anteroposterior mandibular fossa position, vertical condylar inclination, and condylar width and length. The hyperdivergent group showed the significantly highest condylar inclination with the midsagittal plane; anterior and superior positioning of the condyle; smallest anterior, superior, and medial joint spaces; and largest volumetric total joint space relative to the two other groups.

**Conclusions:**

The condyle-fossa position and morphology differ with various vertical facial patterns in individuals with skeletal Class II mandibular retrognathism. These differences could be considered during TMD diagnosis and orthodontic treatment.

**Supplementary Information:**

The online version contains supplementary material available at 10.1186/s12903-022-02174-6.

## Background

The temporomandibular joint (TMJ) is one of the most complex anatomical structures of the human body that has important clinical implications in dentistry. The mandibular condyle is part of the temporomandibular joint structure, and its volume and shape affect the overall stability of the treatment response in orthodontic, orthopedic, orthognathic, and prosthodontic patients [1, 2]. As a result, dental practitioners should consider the condyle’s position and morphology during the treatment procedure.

Skeletal class II malocclusion is considered one of the most prevalent orthodontic problems, accounting for nearly one-third of all orthodontic patients [3]. It can be caused by a variety of factors, although mandibular retrognathism is the most frequent etiologic factor [4]. Many previous studies have found a positive correlation between temporomandibular disorders (TMDs) and abnormal mandibular morphology [5, 6]. In a previous cross-sectional study using lateral cephalometry and magnetic resonance imaging (MRI), disc displacement was associated with the retruded and clockwise-rotated mandible [7].

Several studies have found that the relationship between the condyle and the fossa varies depending on the sagittal and vertical facial shape [8, 9]. Saccucci et al. [10] investigated condylar volume in people with various anteroposterior and vertical skeletal patterns and discovered that hypodivergent subjects have a greater condylar volume than normodivergent or hyperdivergent subjects. Park et al. [11] evaluated condylar morphology in different vertical skeletal patterns and discovered substantial variations in several condylar linear measurements between the hypodivergent and hyperdivergent groups. However, the effects of the craniofacial skeletal patterns on the position and morphology of the TMJ based on the interactive effects of mandibular retrognathism and vertical cephalometric relationships have not been studied and comprehensively understood.

Many previous studies used conventional radiography, MRI, and computed tomography (CT) to evaluate the TMJ structure [12–14]. For bone structure measurements, cone-beam computed tomography (CBCT) has recently been used. It produces images with high resolution and minimal distortion, shorter scanning times, and smaller radiation doses than conventional CT [15, 16]. Measurements of lengths and volumes in several planes are possible with 3D CBCT images, allowing precise diagnoses and predictability of treatment outcomes.

To our knowledge, only a few studies have used 3D CBCT analysis to assess the positional and morphological characteristics of the TMJ in adults with skeletal Class II, and no studies have focused on the case of skeletal Class II with mandibular retrognathism in different vertical skeletal patterns. As a result, the present study aimed to compare the position and morphology of the TMJ in a normal Chinese adult population with mandibular retrognathism in different vertical skeletal patterns.

## Materials and methods

### Sample selection

The research ethics committee of Lanzhou University’s Stomatological Hospital, Lanzhou, Gansu, China, approved this retrospective study (No. LZUKQ-2019-041). The procedures were carried out in conformity with applicable laws and regulations. All patients provided signed consent forms after being given written information.

The sample size was calculated with an alpha value of 0.05 and a power of 80% based on the study conducted by Alhammadi et al. [17], who studied the CBCT 3D analysis of the TMJ in different vertical skeletal facial patterns. They designed superior and anterior joint space data by considering a mean difference of 1.1 mm in superior joint space and 0.5 mm in anterior joint space among groups. As a result, a sample size of 13 or 16 subjects was obtained based on superior or anterior joint space. This sample size was increased to 20 subjects for each group.

In this study, we included 80 adult patients (45 males and 35 females). According to the cephalometric analysis of the Chinese norms [18], subjects with skeletal Class II with a normal sagittal position of the maxilla and mandibular retrognathism (SNA = 82.8° ± 4, A-Nv =  ± 1 ± 2 mm, SNB < 76°, ANB > 5°, and POG-Nv ˂ − 5 mm) were divided into three equal groups (20 subjects each) based on the mandibular angle (SN-MP) and facial height ratio (FHR): hypodivergent group (SN-MP < 27.3° and FHR > 65%), normodivergent group (SN-MP = 32.5° ± 5.2° and FHR = 62–65%), and hyperdivergent group (SN-MP > 37.7° and FHR < 62%), besides 20 subjects for the control group with normal sagittal skeletal relation (SNA = 82.8° ± 4, A-Nv =  ± 1 ± 2 mm, SNB = 80.1° ± 3.9°, ANB = 2.7° ± 2.0°, and POG-Nv = − 2 ± 2 mm) and normal vertical craniofacial dimensions (SN-MP = 32.5° ± 5.2° and FHR = 62–65%; see Table 3).

The following inclusion criteria were applied: (1) aged 18–30 years old, (2) except for the third molar teeth, all permanent teeth have erupted, (3) no history of TMD symptoms, (4) no facial asymmetry or functional mandibular deviations, (5) no surgical history of TMJ or the craniofacial region, (6) no condylar imaging findings of degenerative illness (e.g., subchondral cyst, erosion, and condylar hyperplasia), (7) no orthodontic or orthognathic treatment history, and (8) no skeletal malformation in the craniofacial region.

### Three-dimensional CBCT

The I-CAT Image System (Imaging Sciences International Inc. Hatfield, USA) was used to perform CBCT. We used the following protocol: the field of view (FOV) was 16.0 × 13.0 cm, the setting of the exposure parameter was 120 kV; 18.54 MAs; 8.9 s, the image voxel size was 0.3 mm, maximum occlusal intercuspation, head position standardization, and the Frankfort horizontal plane (FHP) was parallel to the floor. For the 3D analysis of all the linear and angular measures of the TMJ images, we used the Invivo 6/Anatomage dental software program.

**Fig. 1:**
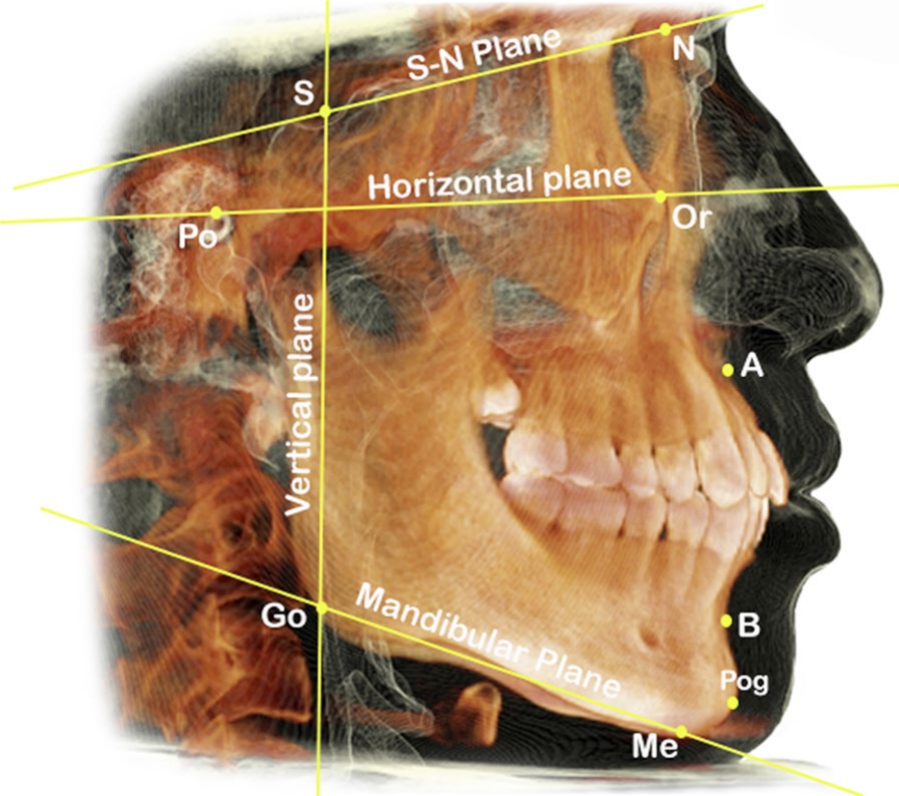
Craniofacial reference planes. Reproduced with modification from Abdulqader et al. [20]

**Fig. 2:**
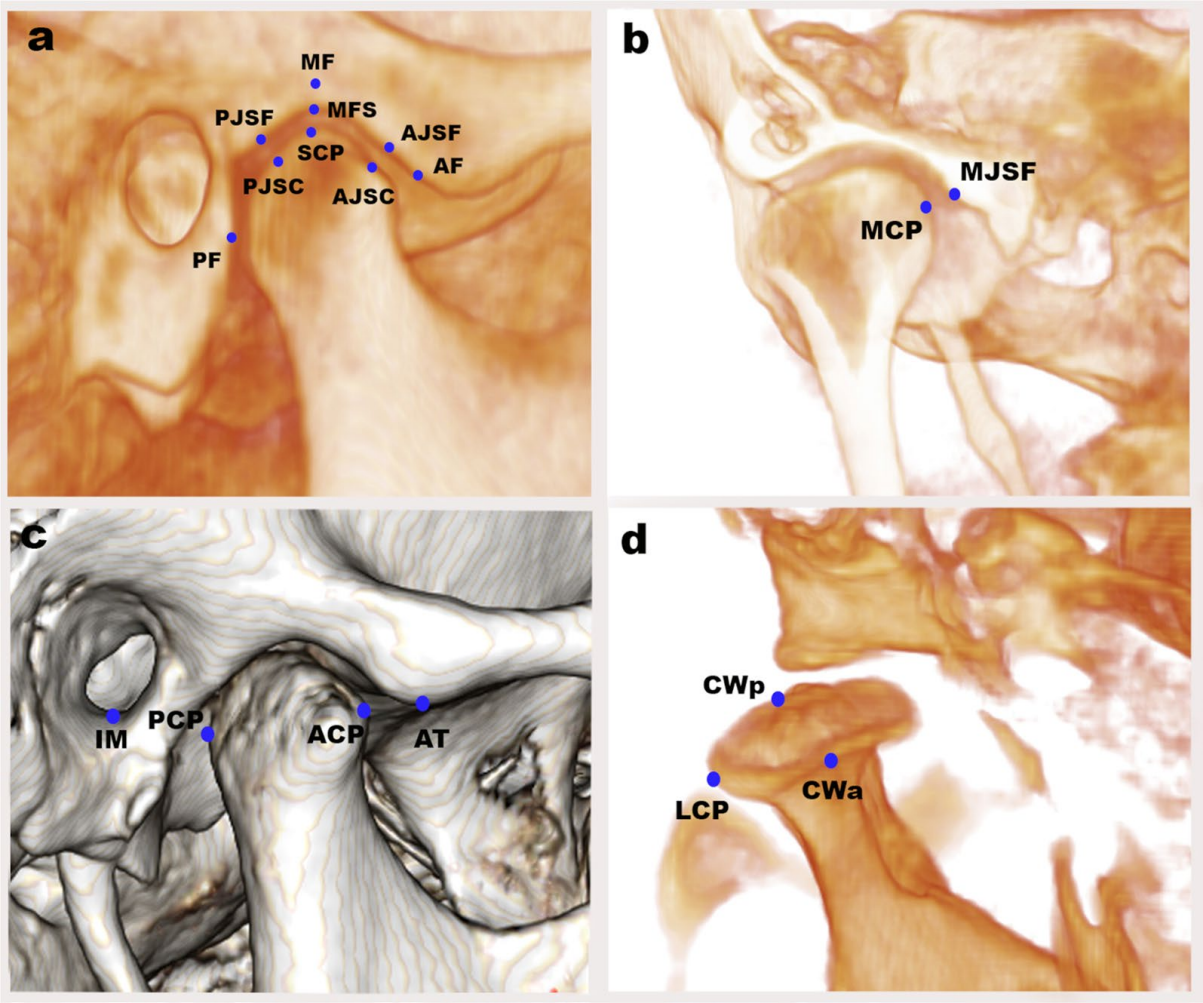
3D temporomandibular joint landmarks: **a, c** sagittal views, **b** coronal view, **d** axial view

The standardized, innovative 3D TMJ analysis method, designed by Alhammadi et al. [19–21], was applied in our study. The craniofacial and TMJ landmarks are shown in Table 1. The reference planes, lines, and 3D measurements of TMJ are shown in Table 2. Craniofacial reference planes are shown in Fig. 1, and the 3D TMJ landmarks are shown in Fig. 2.

**Table 1:** Definitions of 3D skeletal and temporomandibular landmarks used in the study

Landmark	Abb	Definition
*Skeletal landmarks*		
Nasion	N	The most superior and anterior point of the frontonasal suture
Sella	S	The center of the hypophyseal fossa in the middle cranial fossa (Sella turcica)
Basion	Ba	The posterior tip of the skull base, sagittally the most inferior posterior-point of the foramen magnum
Incisive Foramen	IF	The center of incisive foramen centered mediolateral, exists posterior to the central incisors at maxillary mid-palatine
Orbital	Or	The lowest point on the inferior border of the orbit
Porion	Po	The most outer and superior bony point of the external auditory meatus
Gonion	Go	The point of bisecting angle connecting the ramus line and body of mandible line
Menton	Me	The most inferior midpoint of the chin on the mandibular symphysis outline
Subspinale	A	The deepest anterior point in the concavity of the premaxilla
Supramental	B	The deepest anterior point in the concavity of the anterior mandible
Pogonion	Pog	The most anterior point on the mandibular symphysis
*Temporomandibular landmarks*		
Mandibular fossa “Soft tissue point”	MFS	The highest and middlemost point of the soft tissue mandibular fossa
Mandibular fossa “Bony point”	MF	The highest and middlemost point of the bony mandibular fossa
Medial joint space “Tubercle point”	MJSf	The most lateral point of the mandibular fossa medial wall
Superior condylar point	SCP	The most top point of the condylar head
Medial condylar point	MCP	The medial most point of the condylar head
Lateral condylar point	LCP	The lateral most point of the condylar head
Condylar width “Anterior point”	CWa	Axially, the most prominent point anteriorly of the condylar head at the region with the greatest width
Condylar width “Posterior point”	CWp	Axially, the most prominent point posteriorly of the condylar head at the region with the greatest width
Anterior condylar point	ACP	The sagittal most prominent point anteriorly of the condylar head
Posterior condylar point	PCP	The sagittal most prominent point posteriorly of the condylar head
Articular tubercle	AT	The most prominent inferior point of the anterior tubercle
Inferior meatus	IM	The lateral and most inferior point of the external auditory-meatus
Anterior fossa point	AF	The inferior and most anterior point in the inner anterior wall of the glenoid fossa
Posterior fossa point	PF	The inferior and most posterior point in the inner posterior wall of the glenoid fossa, which is parallel to the IM point
Anterior joint space “Mandibular fossa point”	AJSf	The most prominently posterior point of the inner anterior wall of the glenoid fossa nearly at the nearest distance to anterior joint space condylar point
Anterior joint space “Condylar point”	AJSc	The most prominent condyle head’s anterior point nearly at the nearest distance to anterior joint space fossa point
Posterior joint space “Mandibular fossa point”	PJSf	The most prominently anterior point of the inner posterior wall of the glenoid fossa nearly at the nearest distance to posterior joint space condylar point
Posterior joint space “Condylar point”	PJSc	The most prominent condyle head’s posterior point nearly at the nearest distance to posterior joint space fossa point

**Table 2:** Definitions of 3D skeletal and temporomandibular measurements used in the study

Measurements	Abb	Definition
*Reference planes, lines*		
Horizontal plane	HP	A plane extending from right, left porion, and right orbital
Mid-sagittal plane	MSP	A plane constructed by three-point N, BA, and IF
Vertical plane	VP	A plane constructed of sella point and perpendicular to MSP and HP
Mandibular plane	MP	A plane extending from right, left gonion and menton
Nasion vertical line	Nv	A line extending from nasion perpendicular to the Frankfort horizontal plane
Mandibular fossa horizontal plane	MFHL	A plane tangent to the mandibular fossa bony point and parallel to the horizontal plane
TM line	TML	A line determined through the auditory meatus and anterior tubercle
Mandibular fossa line	MFL	A line determined through two bony mandibular fossa MF
Anteroposterior condylar line	ACP–PCP	A line extending from ACP to PCP
Mediolateral condylar line	MCP-LCP	A line extending from MCP to LCD
*Skeletal measurements*		
Anteroposterior position of the maxilla	SNA	The angle formed by the three-point landmarks S, N, and A
Anteroposterior position of the mandible	SNB	The angle formed by the three-point landmarks S, N, and B
Anteroposterior skeletal jaw relation	ANB	The angle formed by the three-point landmarks A, N, and B
Pog-Nv distance	Pog-Nv	The linear distance measured between point Pog and Nv line, determining the anteroposterior position of the chin relative to the nasion vertical line
A-Nv distance	A-Nv	The linear distance measured between point A and Nv line, measuring the anteroposterior position of the maxilla relative to the nasion vertical line
Mandibular angle	SN-MP	The angle formed between the S–N line and mandibular plane Go-Me
Facial height ratio	FHR	The ratio between the posterior facial height (S-Go) and the anterior facial height (N-Me)
*Mandibular fossa dimensions*		
Mandibular fossa height (mm)	MFH	The perpendicular distance between bony MF and TM line
Mandibular fossa width (mm)	MFW	The distance extending horizontally between AF and PF
Inter-fossa distance (mm)	IFD	The distance extending horizontally between right and left MF bony points
Articular eminence height	AEH	The perpendicular distance between AT and MFHL
*Mandibular fossa inclination*		
Mediolateral (°)	MF-MLI	The angle constructed by MF line and VP
Vertical (°)	MF-VI	The angle constructed by MF line and HP
Anteroposterior (°)	MF-API	The angle constructed by MF line and MSP
*Mandibular fossa position*		
Mediolateral (mm)	MF-MLP	The distance extending from the MF bony point to the MSP
Vertical (mm)	MF-VP	The distance extending perpendicularly from the MF bony point to the HP
Anteroposterior (mm)	MF-APP	The distance extending anteroposteriorly from the MF bony point to the VP
*Condylar inclination*		
Mediolateral (°)	MCI	The angle between MCP-LCP and HP
Vertical (°)	VCI	The angle between ACP-PCP and VP
Anteroposterior (°)	APCI	The angle between MCP-LCP and MSP
*Condylar position*		
Mediolateral (mm)	MLCP	The distance extending mediolaterally from MCP to the MSP
Vertical (mm)	VCP	The distance extending vertically from SCP to the HP
Anteroposterior (mm)	APCP	The distance between the ACP and VP
AP condylar joint position (%)	APCJP	The anteroposterior condylar position within the joint according to the formula of Pullinger and Hollender
*Condylar dimension*		
Condylar length (mm)	CL	The mediolateral condylar distance between LCP and MCP
Condylar width (mm)	CW	The anteroposterior condylar distance between ACP and PCP
Condylar height to TM line (mm)	CH	The distance which extends perpendicular from SCP to the TM line
*TMJ space*		
Anterior joint space (mm)	AJS	Closest distance between AJSc and AJSf
Superior joint space (mm)	SJS	Closest distance between SCP and MF
Posterior joint space (mm)	PJS	Closest distance between PJSc and PJSf points
Medial joint space (mm)	MJS	Closest distance between MJSf and MCP
Volumetric total joint space (mm^3^)	VTJS	Total mandibular joint spaces (superior, anterior, and posterior) which are enclosed by the TM line

The condylar position was determined with accuracy and precision using two alternative methods. We assessed the condylar position concerning the craniofacial structure (basal reference planes) in the first method. The second one was by using the formula presented by Pullinger et al. [22]:

$$\frac{P-A}{P+A }\times 100$$ %

where A represents the anterior joint space, and P represents the posterior joint space. The condyle was defined to be in a posterior position if the ratio was less than − 12%. If the ratio was more than + 12%, the condyle was defined to be anteriorly positioned. The condylar position was defined to be concentric when the ratio was within ± 12%.

**Fig. 3:**
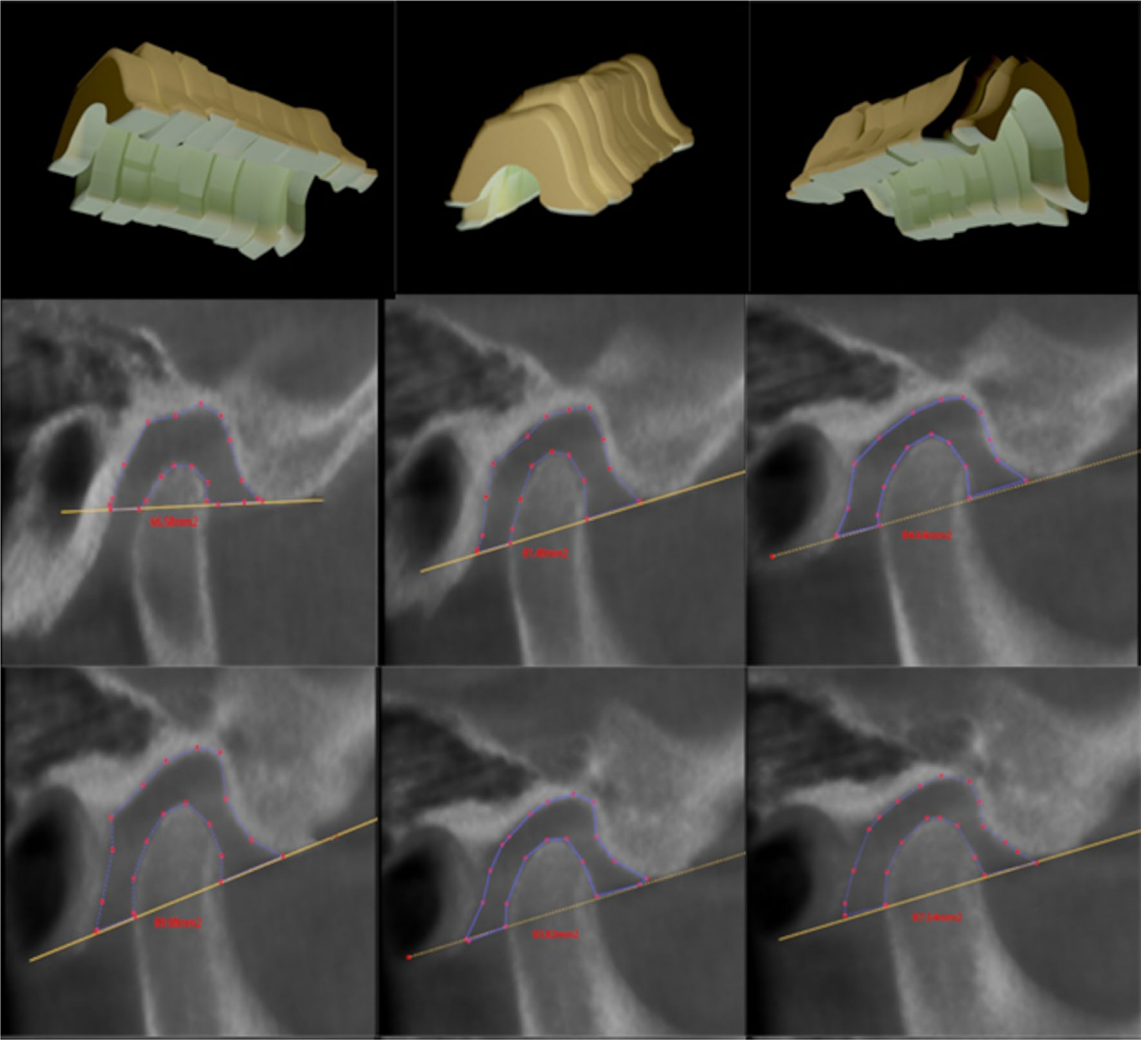
3D volumetric total joint space with 2D identification points. Reproduced with permission from Abdulqader et al. [20]

The TM line, which extends from the inferior point of the articular tubercle (AT) to the inferior point of the auditory meatus (AM), was used to determine the total joint space. Previous research has been published [20], and we followed their method of measuring the volumetric joint space via cubic 3D analysis of the total area by sectioning the whole joint space into six sections. The width of each section was 1.5 mm, as shown in Fig. 3. We then calculated the space with the equation of sigma volume ($v\cong \sum_{k=1}A\left({x}_{\dot{I}}\right)\Delta x$). Two different observers re-analyzed the cases within 2 weeks to ensure intra- and inter-examiner reliability of the measurements.

### Statistical analysis

IBM SPSS Statistics software, version 24 for Windows (IBM Corp., Armonk, NY, USA), was used to perform the statistical analysis. Intra-class correlation coefficient (ICC) and Bland–Altman plots were used to determine the TMJ landmark’s reproducibility and reliability. For each of the 80 subjects, a descriptive statistic of the mean and standard deviation (SD) is provided, with the result’s significance set at *P* < 0.05. The Shapiro–Wilk test was used to check the normal distribution of the data. We used one-way ANOVA and the Tukey post hoc test to compare the mean values between the groups.

## Results

Eighty patients achieved the inclusion and exclusion criteria (45 males and 35 females), with an average age of 23.36 ± 3.11 years. The anteroposterior and vertical skeletal relationships of the selected sample are presented in Table 3. Intra- and inter-observer reliabilities for X, Y, and Z coordinates of all the TMJ landmarks were excellent with ICC [see Additional file 1]. Bland–Altman analysis demonstrated very good intra- and inter-observer agreement between coordinates for all TMJ landmarks [see Additional file 2]. The descriptive statistics and significant (*P*) values of ANOVA and Tukey tests for the TMJ measurements of all groups are presented in Table 4.

**Table 3:** Distribution of subjects among groups

Variables	Groups			
	Control	Hypodivergent	Normodivergent	Hyperdivergent
Patient (n)	20	20	20	20
Age (year)	22.15 ± 2.46	22.65 ± 3.51	24.75 ± 2.61	23.90 ± 3.26
*SEX*				
Male	11	13	12	9
Female	9	7	8	11
SNA (°)	82.46 ± 1.81	81.16 ± 1.40	80.94 ± 1.18	80.92 ± 1.11
SNB (°)	79.98 ± 1.47	74.54 ± 0.60	73.98 ± 1.17	72.24 ± 1.24
ANB (°)	2.48 ± 0.87	6.63 ± 1.40	6.96 ± 1.45	8.68 ± 1.54
POG-Nv (mm)	− 2.16 ± 1.82	− 6.29 ± 3.41	− 7.83 ± 4.33	− 13.50 ± 3.99
A-Nv (mm)	1.45 ± 1.41	1.84 ± 0.91	1.68 ± 1.44	1.64 ± 1.40
SN-MP (°)	32.15 ± 2.14	24.95 ± 1.41	34.31 ± 2.16	45.13 ± 2.50
FHR (%)	63.90 ± 1.21	71.45 ± 1.64	64.15 ± 1.27	57.74 ± 1.55

**Table 4:** Descriptive statistics and significant *P*-values of ANOVA and Tukey post hoc tests for the measurements of the temporomandibular joint in the control group and skeletal Cl II with mandibular retrognathism in different vertical skeletal patterns

Variable	Control group		Hypodivergent group		Normodivergent group		Hyperdivergent group		ANOVA	Multiple Comparison Tukey's HSD test (*P* Value)					
										Comparison of all groups to control group			Comparison class II among different skeletal pattern		
	M	SD	M	SD	M	SD	M	SD	*P*-Value	Control/Hypo-	Control/Normo-	Control/Hyper-	Hypo-/Normo-	Normo-/Hyper-	Hypo-/Hyper-
*Mandibular fossa measurement*															
MFH (mm)	9.24	1.15	9.01	1.02	9.38	0.97	9.69	1.08	0.238	NS	NS	NS	NS	NS	NS
MFW (mm)	16.67	1.41	16.41	1.04	16.53	0.95	16.57	2.50	0.966	NS	NS	NS	NS	NS	NS
IFD (mm)	102.88	5.71	103.63	4.24	103.26	4.62	103.10	2.98	0.960	NS	NS	NS	NS	NS	NS
AEH (mm)	8.04	1.42	8.47	1.15	8.10	1.43	8.14	1.37	0.746	NS	NS	NS	NS	NS	NS
MFMLP (mm)	51.45	2.89	51.92	2.34	51.62	2.31	51.80	1.33	0.922	NS	NS	NS	NS	NS	NS
MF-VP (mm)	1.90	0.87	2.61	0.73	1.89	0.87	1.19	0.58	0.000***	0.026	NS	0.024	0.023	0.026	0.000
MF-APP (mm)	9.48	1.93	11.13	2.21	9.56	1.86	7.84	1.45	0.000***	0.035	NS	0.036	0.049	0.025	0.000
MF-MLI (°)	1.12	0.70	0.85	0.74	1.06	0.47	1.40	0.78	0.080	NS	NS	NS	NS	NS	NS
MF-VI (°)	0.40	0.28	0.42	0.34	0.43	0.26	0.58	0.41	0.303	NS	NS	NS	NS	NS	NS
MF-API (°)	88.63	0.85	88.93	0.71	88.77	0.46	88.25	1.17	0.145	NS	NS	NS	NS	NS	NS
*Mandibular condyle measurement*															
MCI (°)	9.96	3.92	8.58	4.31	10.82	4.20	14.00	6.26	0.005**	NS	NS	0.043	NS	NS	0.003
VCI (°)	64.20	7.22	55.69	4.40	67.09	5.12	71.85	8.90	0.000***	0.001	NS	0.003	0.000	NS	0.000
APCI (°)	73.66	5.62	74.75	4.75	72.75	5.99	68.60	6.81	0.008**	NS	NS	0.038	NS	NS	0.007
VCP (mm)	2.83	1.51	3.53	1.12	2.13	1.52	1.33	0.72	0.000***	NS	NS	0.002	0.004	NS	0.000
APCP (mm)	7.41	2.56	8.35	2.76	6.89	1.98	4.85	1.99	0.000***	NS	NS	0.005	NS	0.038	0.000
MLCP (mm)	51.34	2.66	51.74	2.44	51.14	2.01	51.14	1.88	0.820	NS	NS	NS	NS	NS	NS
APCJP (%)	− 2.45	17.37	4.78	15.00	3.56	12.12	14.27	13.44	0.005**	NS	NS	0.003	NS	0.007	0.037
CL (mm)	17.89	1.61	19.36	1.68	18.48	1.45	16.25	1.92	0.000***	0.028	NS	0.025	NS	0.001	0.000
CW (mm)	7.28	0.78	8.03	0.83	7.63	1.06	6.52	0.80	0.000***	0.041	NS	0.035	NS	0.001	0.000
CH (mm)	4.63	1.19	4.76	0.82	5.32	1.08	6.62	0.85	0.000***	NS	NS	0.000	NS	0.007	0.000
TMJ space															
AJS (mm)	2.63	0.53	2.34	0.51	2.39	0.61	1.92	0.37	0.000***	NS	NS	0.000	NS	0.023	NS
SJS (mm)	3.47	0.57	3.45	0.73	3.36	0.73	2.45	0.47	0.000***	NS	NS	0.000	NS	0.000	0.000
PJS (mm)	2.56	0.76	2.55	0.43	2.56	0.58	2.57	0.46	1.000	NS	NS	NS	NS	NS	NS
MJS (mm)	2.84	0.54	2.82	0.58	2.71	0.58	2.20	0.49	0.001**	NS	NS	0.003	NS	0.022	0.004
VTJS (mm3)	282.41	32.47	277.53	29.91	287.85	41.39	316.17	45.79	0.008**	NS	NS	0.030	NS	NS	0.009

*M*, mean value; *SD*, standard deviation; *ANOVA*, Analysis of Variance; *NS*, Not significant; *Hypo*-, Hypodivergent; *Normo*-, Normodivergent; *Hyper*-, Hyperdivergent

*P < 0.05; **P < 0.01; ***P < 0.001

For the mandibular fossa (MF) measurements, no statistically significant differences were found between the groups, except the MF position, in which the hyperdivergent group showed significantly superior and anterior positioning of the MF (1.19 ± 0.58 and 7.84 ± 1.45 mm, respectively). In comparison, the hypodivergent group showed a significantly inferior and posterior positioning of the MF (2.61 ± 0.73 and 11.13 ± 2.21 mm, respectively).

Regarding mandibular condyle measurements, the hyperdivergent group showed the significantly highest inclination with VP (71.85° ± 8.90°) and the lowest inclination with MSP (68.60° ± 6.81°). By contrast, the hypodivergent group showed the significantly lowest inclination with VP (55.69° ± 4.40°).

Regarding the condylar position, the hyperdivergent group showed a statistically significant superior and anterior condyle positioning of 1.33 ± 0.72 and 4.85 ± 1.99 mm, respectively. For the percentage of posterior to anterior joint spaces, the hyperdivergent group showed the statistically significant highest ratio of anteroposterior condylar joint position (APCJP; 14.27% ± 13.44%), which defined the condyle to be in an anterior position in this group.

For the condylar dimensions, the hypodivergent group showed the highest mediolateral and anteroposterior dimensions of 19.36 ± 1.68 and 8.03 ± 0.83 mm, respectively. By contrast, the hyperdivergent group showed the lowest mediolateral and anteroposterior dimensions of 16.25 ± 1.92 and 6.52 ± 0.80 mm, respectively, with the highest condylar height of 6.62 ± 0.85 mm.

Concerning TMJ spaces, the hyperdivergent group showed the significantly smallest anterior, superior, and medial joint spaces of 1.92 ± 0.37, 2.45 ± 0.47, and 2.20 ± 0.49 mm, respectively. In addition, the volumetric total joint space (VTJS) showed a significant increase (316.17 ± 45.79 mm^3^) in the hyperdivergent group compared with the other groups.

The normodivergent group has some condyle-fossa morphology and position variations, but these variations are not statically significant as the changes found in the other two Class II groups.

## Discussion

The morphology of the TMJ varies between individuals. Various variables can influence this morphology, including the functional loads; this might be due to the close relationship between form and function, which differs between subjects with different malocclusion types [23]. Many studies have found an association between TMDs and abnormal mandibular morphology, and some of these studies reported the presence of a positive correlation between TMDs and mandibular retrognathism [7, 24]. Besides, the maximum occlusal force and masticatory muscle activity are known to be influenced by the vertical facial pattern [25]. Internal derangement was shown to be more common in patients with the hyperdivergent skeletal pattern, according to Stringert and Worms [26].

Knowledge of the condyle-glenoid fossa relationship in three planes may help specialists detect the onset of degenerative joint disorders or identify problems that have already developed, allowing for better treatment planning [27]. Therefore, clinical interpretations combined with the correct identification of these values can be critical for diagnosis and treatment planning in the various skeletal relationships.

The current study used CBCT to assess TMJ morphology as it is considered an ideal tool for osseous assessment of the anatomic structures related to craniomandibular articulation without superimposition and distortion for better diagnosis and treatment planning [28, 29].

This study showed that the MF in the hyperdivergent group was superior-anteriorly positioned, whereas it was inferior-posteriorly positioned in the hypodivergent group. The MF’s vertical position may influence face morphology, and some investigations revealed a superior position to the high angle class and a low position to the low angle class [30]. The position of the MF differs depending on the dentoskeletal pattern, and it is suggested to be a significant factor in the development of malocclusion; consequently, the position of the MF should be evaluated during diagnosis [31].

The current study found that the hyperdivergent group had the lowest condylar inclination with the mid-sagittal plane, which may indicate that the condyle is more anteriorly situated in this group. The vertical condylar inclination, representing the anterior and posterior condylar inclination, increased in the hyperdivergent group, which showed more posterior rotation of the condyle, and a decrease in the hypodivergent group, which showed the more anterior rotation of the condyle. The current study’s findings can be explained by Gail Burke et al.’s [32] study, which used pre-orthodontic lateral cephalograms and tomograms of 136 preadolescent Class II patients to investigate the association between skeletal growth patterns and condyle glenoid fossa relation. They stated that patients with vertical facial morphologic features have posteriorly angled condyle, whereas patients with horizontal facial morphology have anteriorly angled condyle. Goyal et al. [33] found a similar result: “Posterior inclination of the condyle is evident in typical long face syndrome, while the anterior inclination of the condyle is present in classic short face syndrome.”

The condylar position was evaluated by two separate approaches. The first approach was based on the dependent planes (MSP, HP, and VP). Regarding the anteroposterior condylar position relative to the vertical plane, the current study showed that the patients in the hyperdivergent group had more anteriorly positioned condyle than those in the normodivergent and hypodivergent groups. Anterior or posterior condylar position may have a direct impact on facial morphology. Bjork [34] discovered that patients with a high-angle condyle usually grow backward, resulting in an anterior condylar position. Furthermore, the other studies found that patients with a hyperdivergent facial pattern have more anteriorly positioned condyle than those with a normal or low angle [9, 35]. Regarding vertical condylar position relative to the horizontal plane, our study found a statistically significant superior condyle position in the hyperdivergent group, and this result was similar to previous studies’ findings [11, 27, 32].

The second approach involved determining the concentric position of the mandibular condyle in the glenoid fossa by calculating the ratio between the anterior and posterior joint spaces using the Pullinger formula. The condyle and fossa have a normal relation, as the condyle is in a concentric position to the fossa, which is frequently reported in asymptomatic individuals [22, 36]. TMJ dysfunction has been related to non-concentric condyle-fossa relationships [36–38]. The current study found that the hyperdivergent group showed the statistically significant highest ratio of APCJP, which meant that the condyle was in an anterior position in this group. Therefore, the mandibular condyle appeared to be non-concentric to their articular fossae in the hyperdivergent group. Some investigations also used the same methodologies to evaluate the condylar concentricity in people with varied vertical skeletal patterns. They also found that the mandibular condyle is positioned more anteriorly in the hyperdivergent group, which agrees with our study’s findings [9, 35].

Concerning TMJ spaces, the hyperdivergent group had the smallest anterior, superior, and medial joint spaces than the other groups. In the hyperdivergent group, the low medial joint space may be due to the medial condylar position. The hyperdivergent skeletal pattern is related to more anteriorly positioned condyles, as seen by this group’s much smaller anterior joint space. The sagittal growth of the condyle in the hyperdivergent skeletal pattern [34], which results in backward growing rotation for the mandible and backward inclination for the condylar head [39], may result in a superiorly positioned condyle and a smaller superior joint space since pre-adolescence [11, 32, 40], so the hyperdivergent profile has a smaller superior joint space than the hypodivergent one.

Regarding condylar parameters, the hypodivergent group showed the significantly highest mediolateral and anteroposterior dimensions, whereas the hyperdivergent group showed the significantly lowest mediolateral and anteroposterior dimensions; this finding was consistent with some previous studies [11, 35, 40, 41]. However, some recent studies have found that the condylar width in adolescents does not differ significantly across various vertical skeletal patterns [42]. The variations could be due to differences in participant number and ages and the descriptions of each landmark. Low angle subjects have a higher condylar volume than normal and high angle subjects [10, 43]. Given that the condyle and fossa are well fitted, the occlusal changes are primarily supported by the large condyle and are regarded as resistant to displacement. The small condyle may not provide adequate support for the occlusal alterations and can frequently be displaced because it makes stable component fitting difficult [13, 44]. The changes that appeared in the TMJ parameters varied greatly in the hyperdivergent group due to the complexity of the facial skeletal structure. It may be due to a combination of horizontal and vertical changes of the mandible. As a result, the positional muscle changes that lead to TMD may occur.

The present study showed a significant increase of VTJS in the hyperdivergent group compared with that in the control and hypodivergent groups. The increase in the volumetric joint space reflects a reduction in the condylar volume. In general, mandibular condyle that shows changed morphologies such as reduced volume can strongly indicate TMDs [45]. This assessment can be clinically valuable in diagnosing TMJ in patients with malocclusion and without pain or TMJ dysfunction [2].

In comparison with the normal group, mandibular retrognathism in the class II normodivergent facial pattern group presented a few variations in condyle-fossa morphology and position. However, these variations were not statically significant as the changes were found in the two other Class II groups, especially in the hyperdivergent facial pattern group. These results may be due to the fact that the antero-posterior (AP) position/size of the mandible in the Class II normodivergent facial pattern group had a weaker influence on the measurements than the combination of sagittal and vertical facial characteristics.

This investigation had limitations as only CBCT images were used, so the articular disc could not be assessed. As a result, asymptomatic patients with internal derangement were possibly included in this study. In future research, evidence derived from additional diagnostic methods, including MRI examinations, may be required to rule out TMD patients with disc displacement. Furthermore, the number of patients was insufficient due to the limitations of the retrospective study design. A well-designed prospective study can overcome this problem.

## Conclusion

Significant condyle-fossa morphology and position variations were found in patients with skeletal Class II mandibular retrognathism and various vertical facial types. The TMJ morphology affects patients with skeletal Class II mandibular retrognathism with a hyperdivergent skeletal pattern more than those in the other groups. The knowledge of regular condyle-fossa variations caused by the vertical skeletal patterns of the patients with skeletal Class II mandibular retrognathism could be helpful in the diagnosis of TMJ pathologies, useful for understanding TMDs, and considered during orthodontic treatment.

## Supplementary Information

**Additional file 1.** Intraclass correlation coefficient (ICC) of X, Y, and Z coordinates of the TMJ landmarks in Intra and Inter-observer reliability.

**Additional file 2.** Bland-Altman plots in Intra and Inter-observer reliability for the TMJ landmarks.

## Data Availability

All data and materials are available from the Orthodontics Department of Stomatology School, Lanzhou University, China. Please get in touch with the corresponding authors for any requests.

## Abbreviations

TMJ: Temporomandibular joint
CBCT: Cone-beam Computed Tomography
3D: Three dimensions
CT: Computed tomography
MRI: Magnetic resonance imaging
ICC: Intra-class correlation coefficient
