# Supplementary material for: Comprehensive three-dimensional positional and morphological assessment of the temporomandibular joint in skeletal Class II patients with mandibular retrognathism in different vertical skeletal patterns
Source: BMC Oral Health. 2022 Apr 28;22:149. doi: 10.1186/s12903-022-02174-6 (PMC9052647; doi:10.1186/s12903-022-02174-6)
Supplement: Supplementary file 1 — Additional file 1. Intraclass correlation coefficient (ICC) of X, Y, and Z coordinates of the TMJ landmarks in Intra and Inter-observer reliability. [file 12903_2022_2174_MOESM1_ESM.pdf]

ICC of X, Y, and Z coordinates of the TMJ landmarks in Intra- and Inter-observer reliability

Landmark	Intra-class correlation coefficient (ICC)					
	Intra-Observer reliability			Inter-Observer reliability		
	X	Y	Z	X	Y	Z
MFS	0.984	0.982	0.962	0.973	0.980	0.959
MF	0.989	0.987	0.974	0.986	0.974	0.963
MJSf	0.978	0.972	0.969	0.973	0.961	0.963
SCP	0.984	0.980	0.963	0.960	0.979	0.960
MCP	0.970	0.984	0.983	0.969	0.986	0.971
LCP	0.975	0.968	0.980	0.980	0.984	0.980
CWa	0.985	0.980	0.985	0.977	0.978	0.982
CWp	0.993	0.988	0.971	0.965	0.988	0.973
ACP	0.991	0.989	0.992	0.980	0.988	0.982
PCP	0.990	0.994	0.994	0.991	0.951	0.987
AT	0.988	0.996	0.987	0.992	0.993	0.978
IM	0.978	0.987	0.985	0.983	0.988	0.988
AF	0.983	0.977	0.993	0.989	0.984	0.992
PF	0.996	0.991	0.970	0.996	0.990	0.980
AJSf	0.982	0.995	0.969	0.994	0.994	0.983
AJSc	0.981	0.992	0.971	0.980	0.992	0.991
PJSf	0.987	0.979	0.982	0.983	0.977	0.983
PJSc	0.970	0.989	0.985	0.957	0.993	0.992
