# Supplementary material for: Comprehensive three-dimensional positional and morphological assessment of the temporomandibular joint in skeletal Class II patients with mandibular retrognathism in different vertical skeletal patterns
Source: BMC Oral Health. 2022 Apr 28;22:149. doi: 10.1186/s12903-022-02174-6 (PMC9052647; doi:10.1186/s12903-022-02174-6)

Bland Altman Plot for "Point MFS"

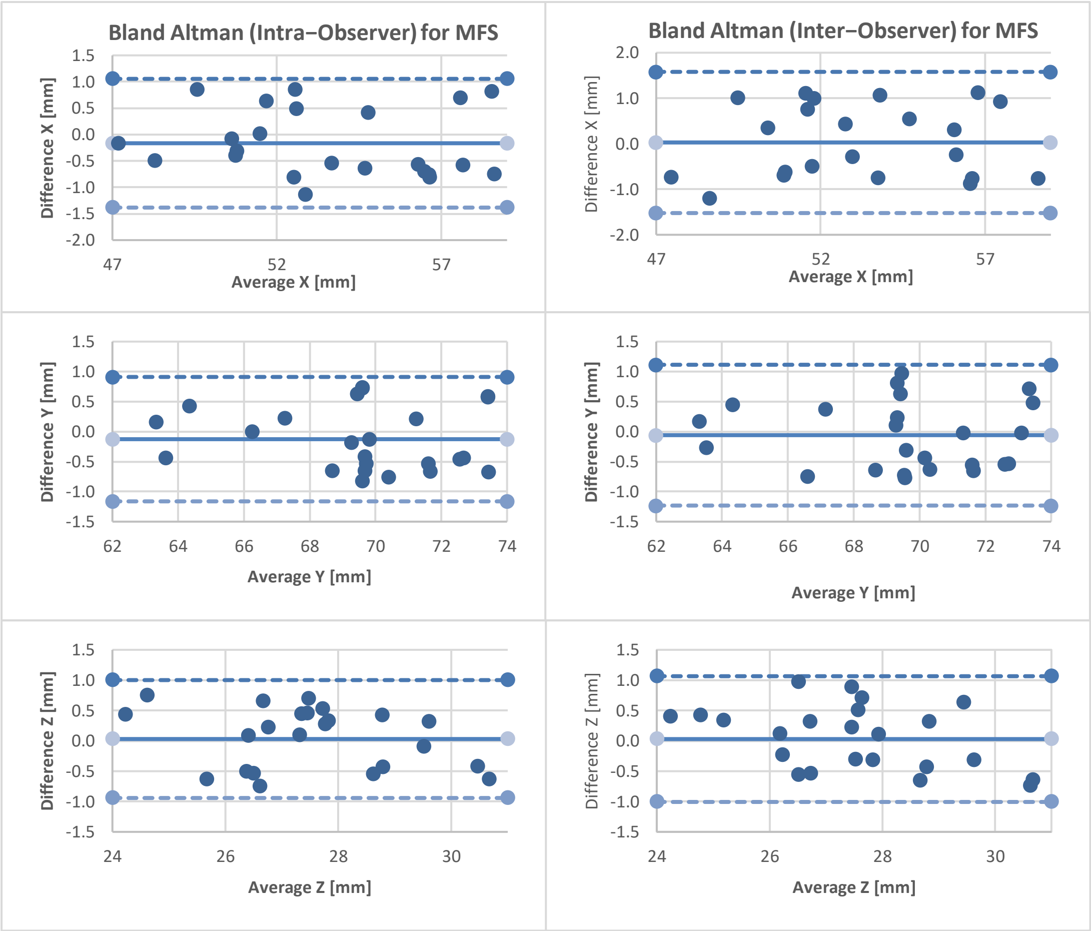

Bland Altman Plot for "Point MF"

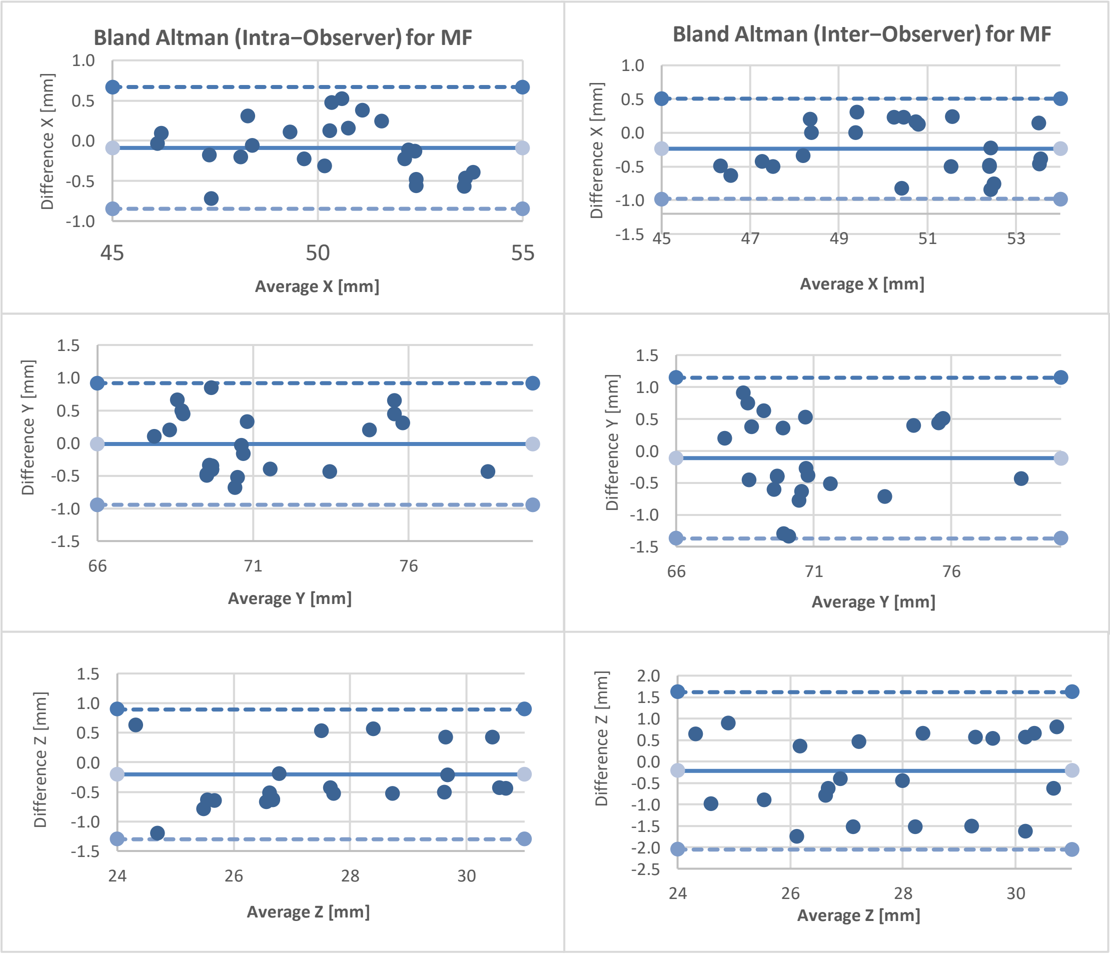

Bland Altman Plot for "Point MJSf "

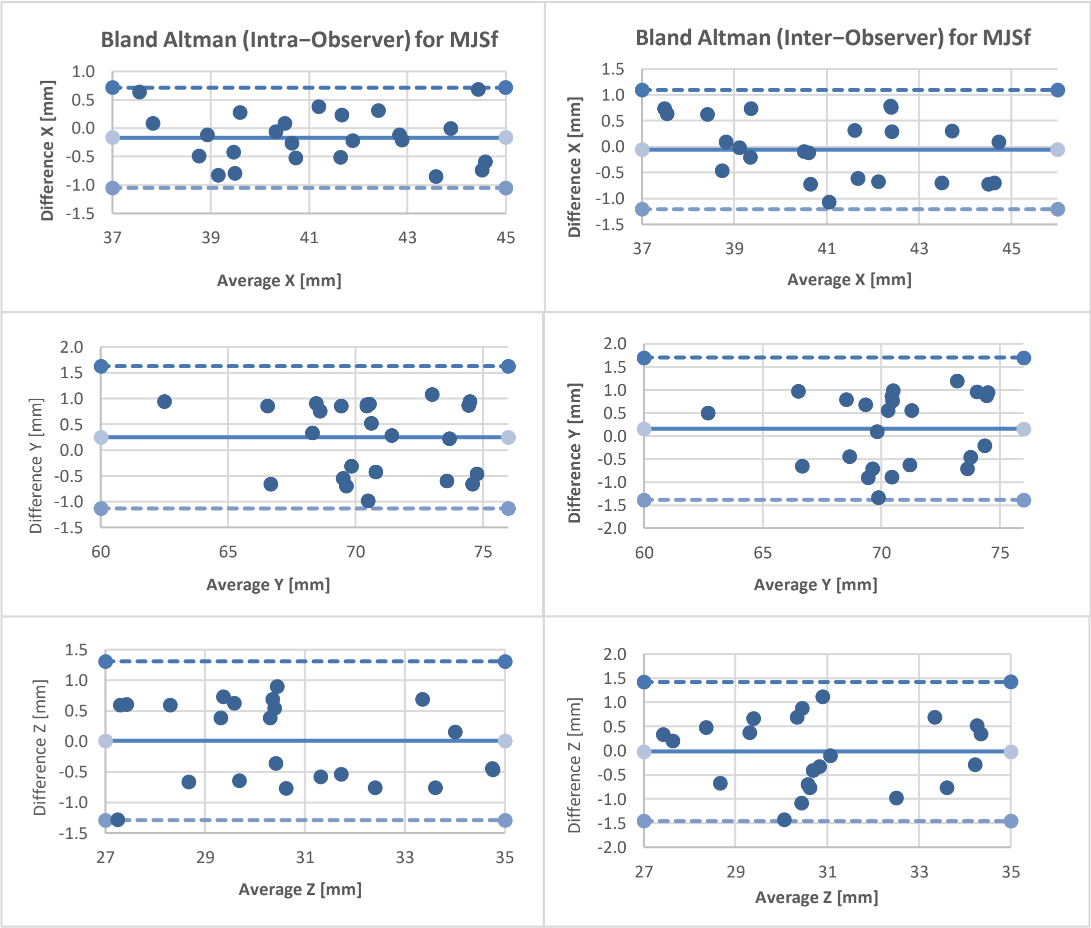

Bland Altman Plot for "Point SCP"

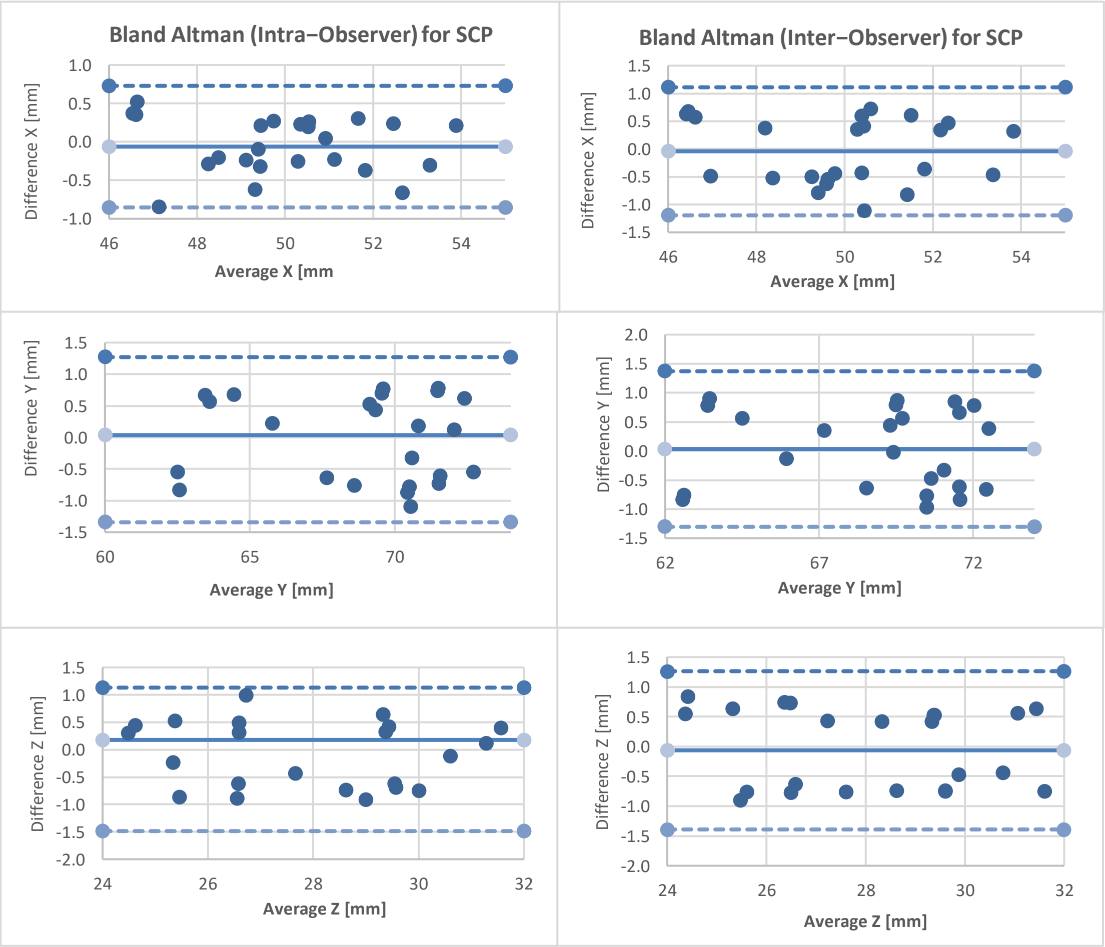

Bland Altman Plot for "Point MCP"

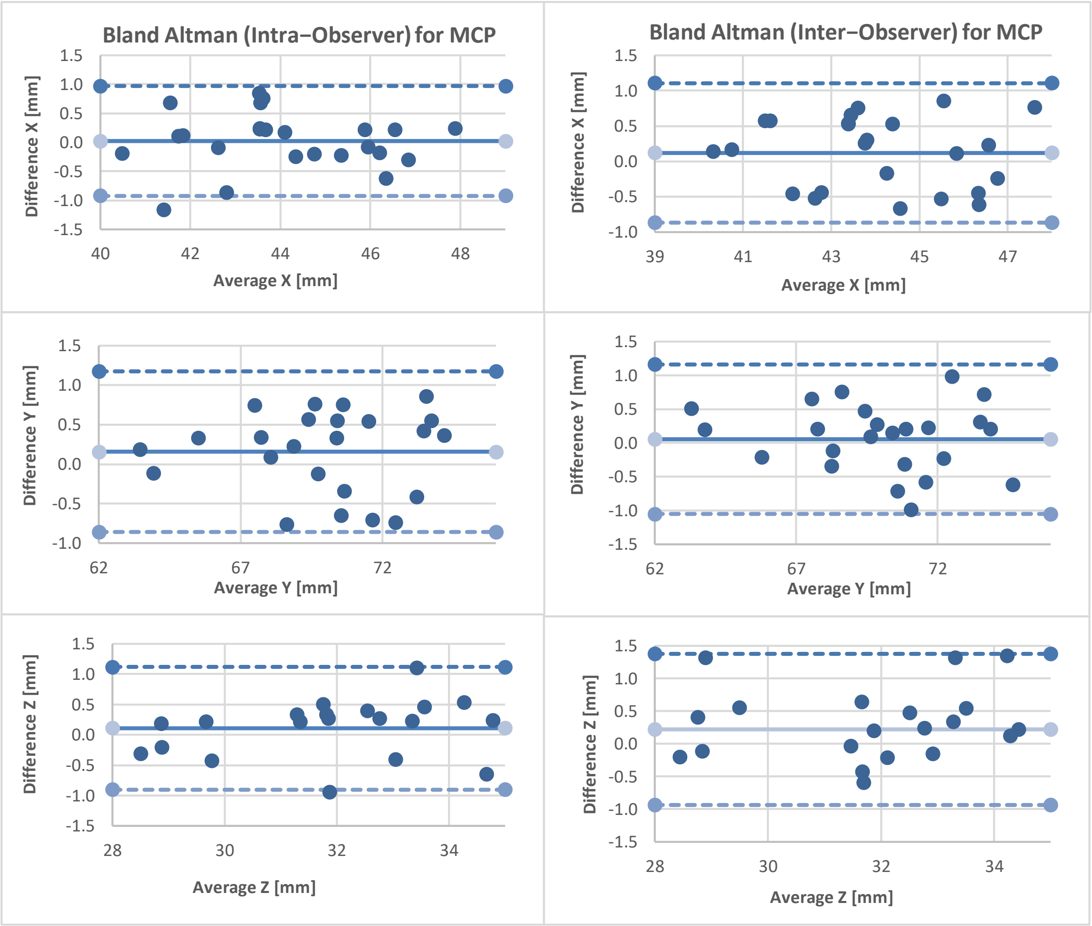

Bland Altman Plot for "Point LCP"

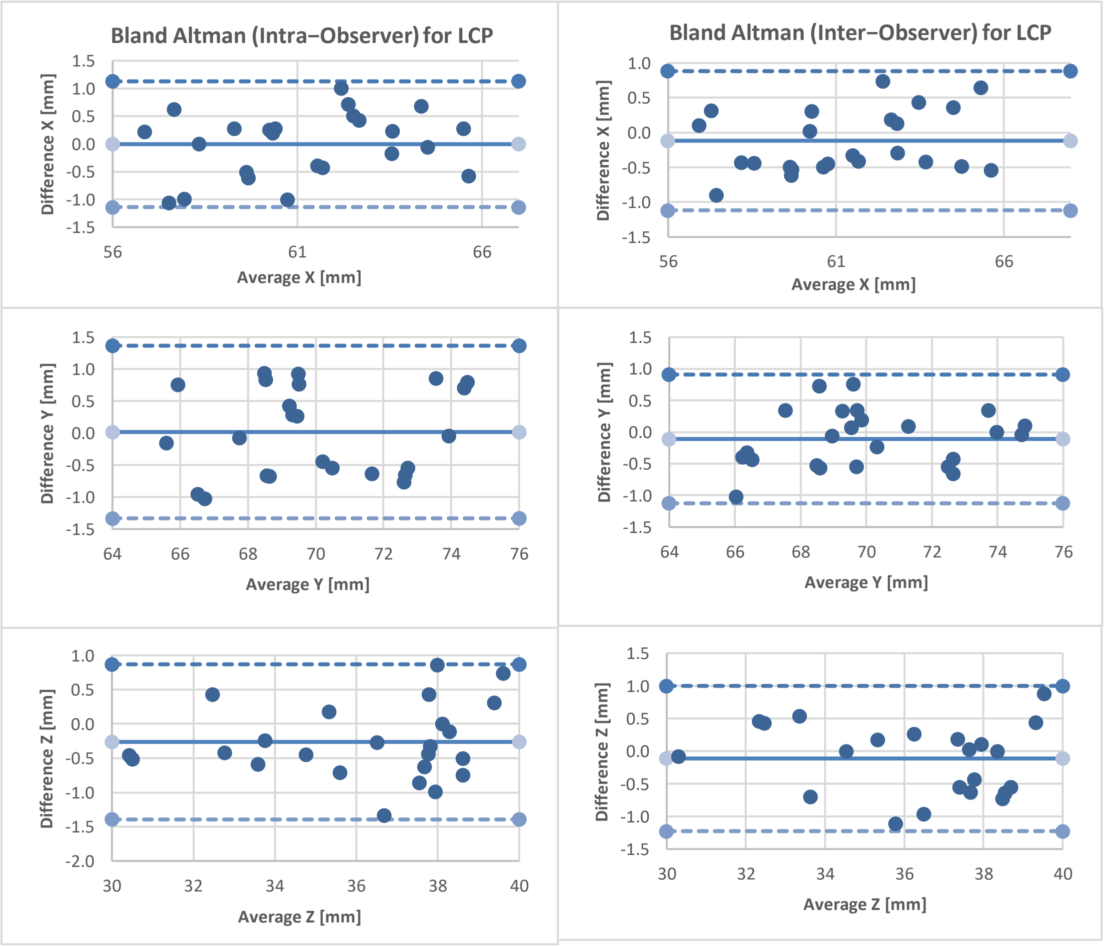

Bland Altman Plot for "Point CWa"

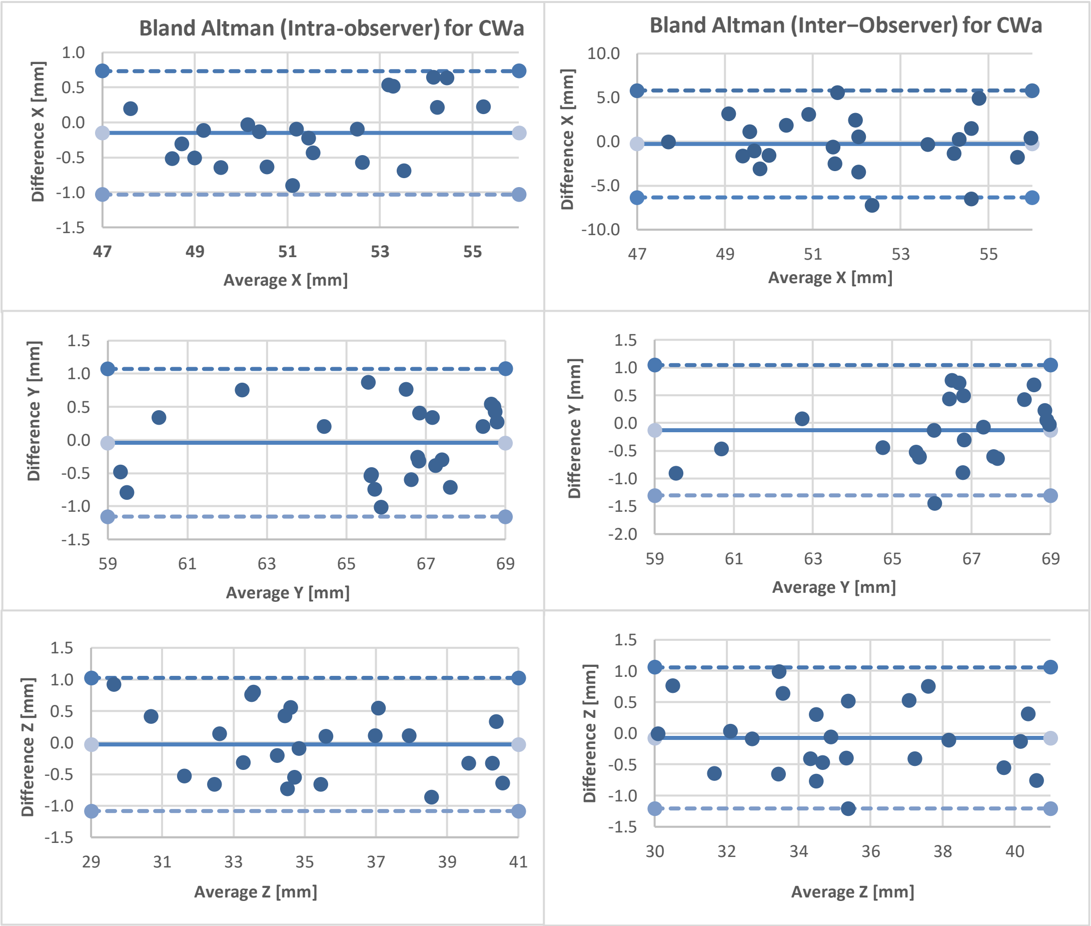

Bland Altman Plot for "Point CWp"

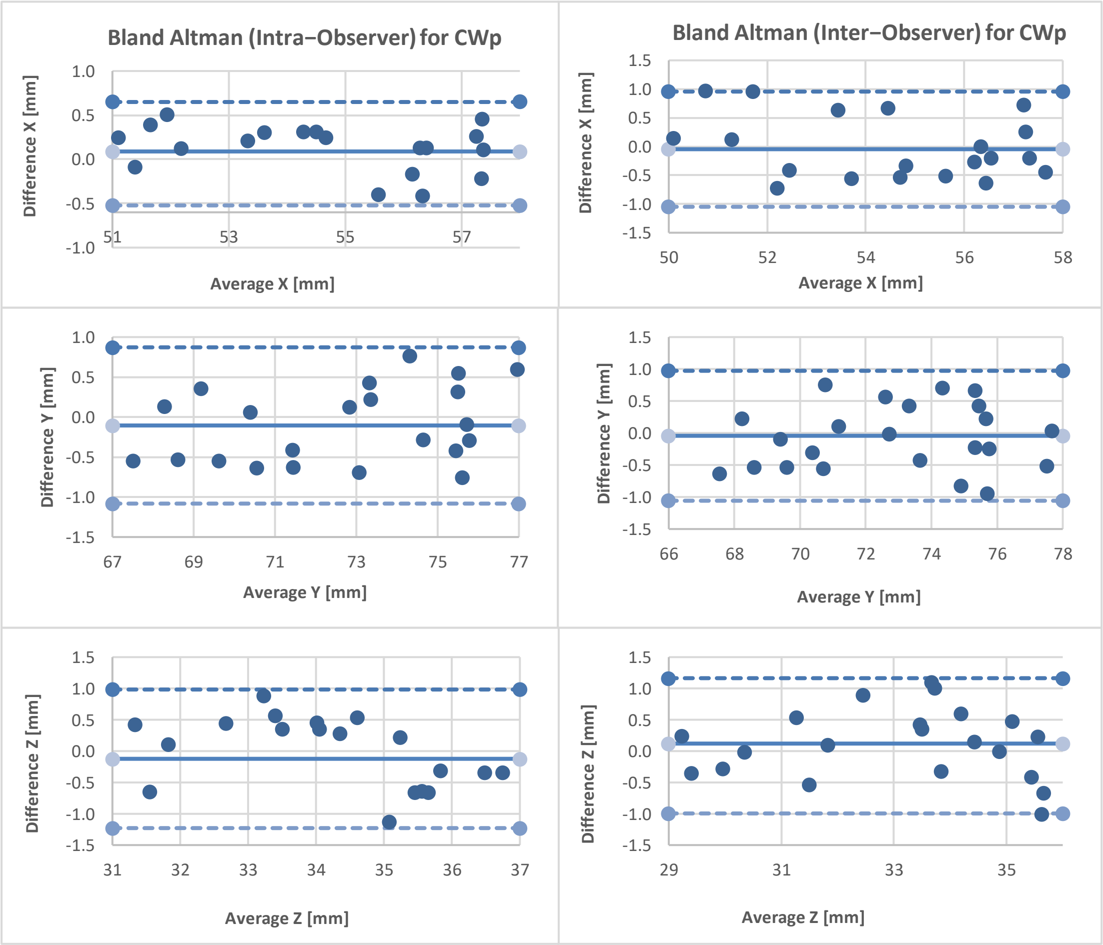

Bland Altman Plot for "Point ACP"

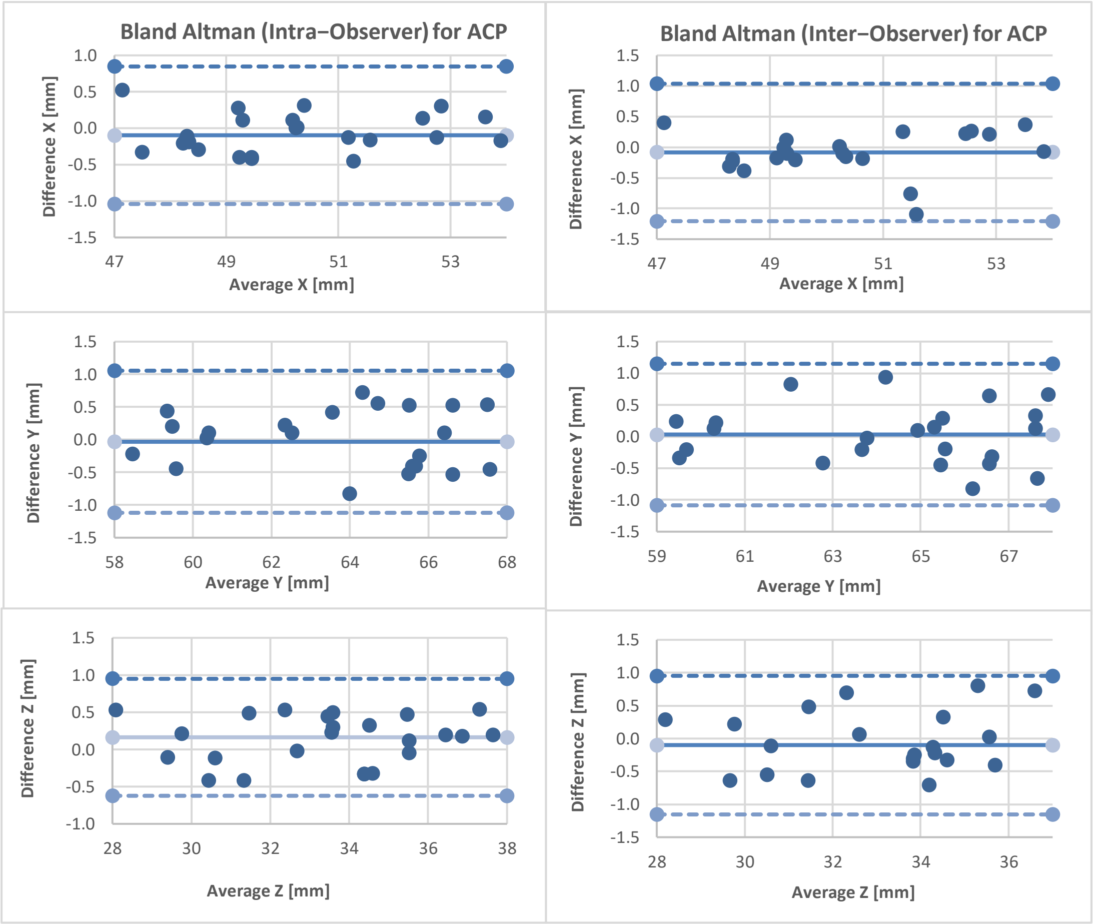

Bland Altman Plot for "Point PCP"

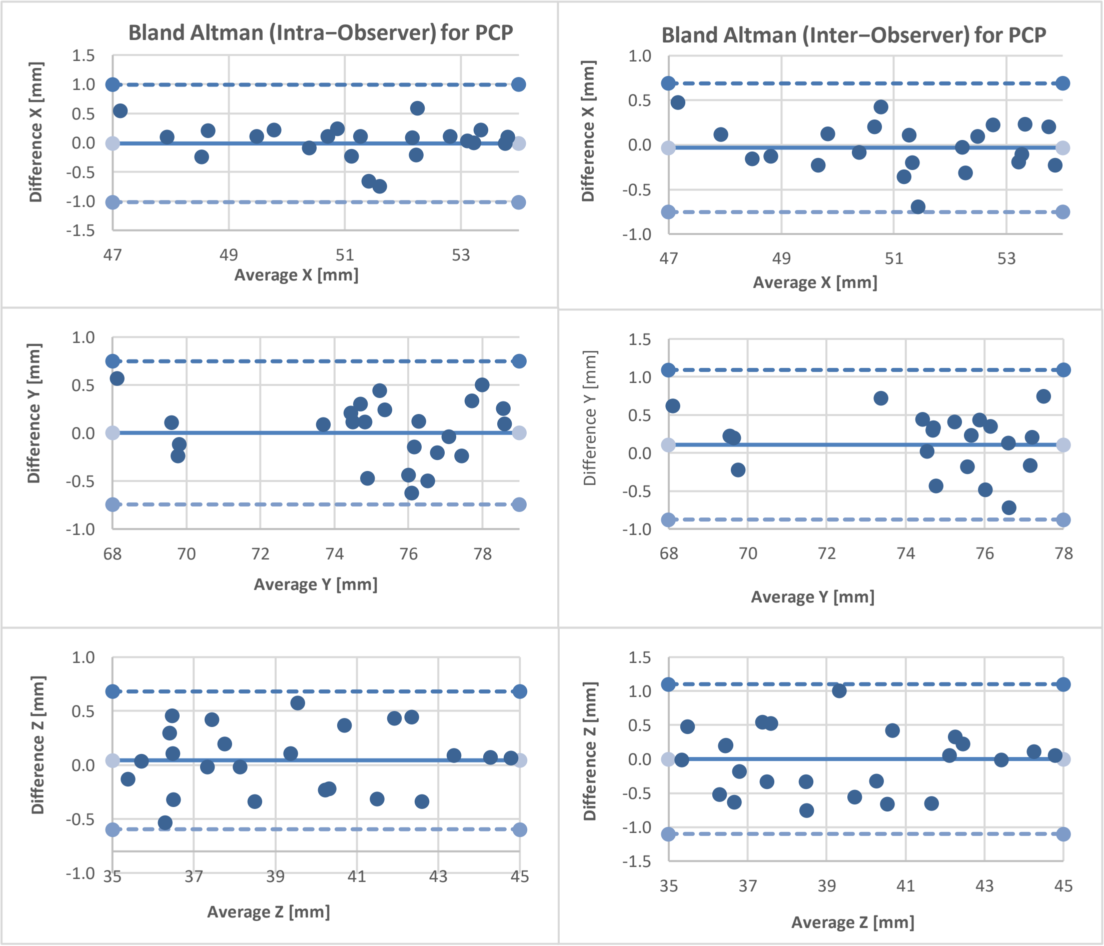

Bland Altman Plot for "Point AT"

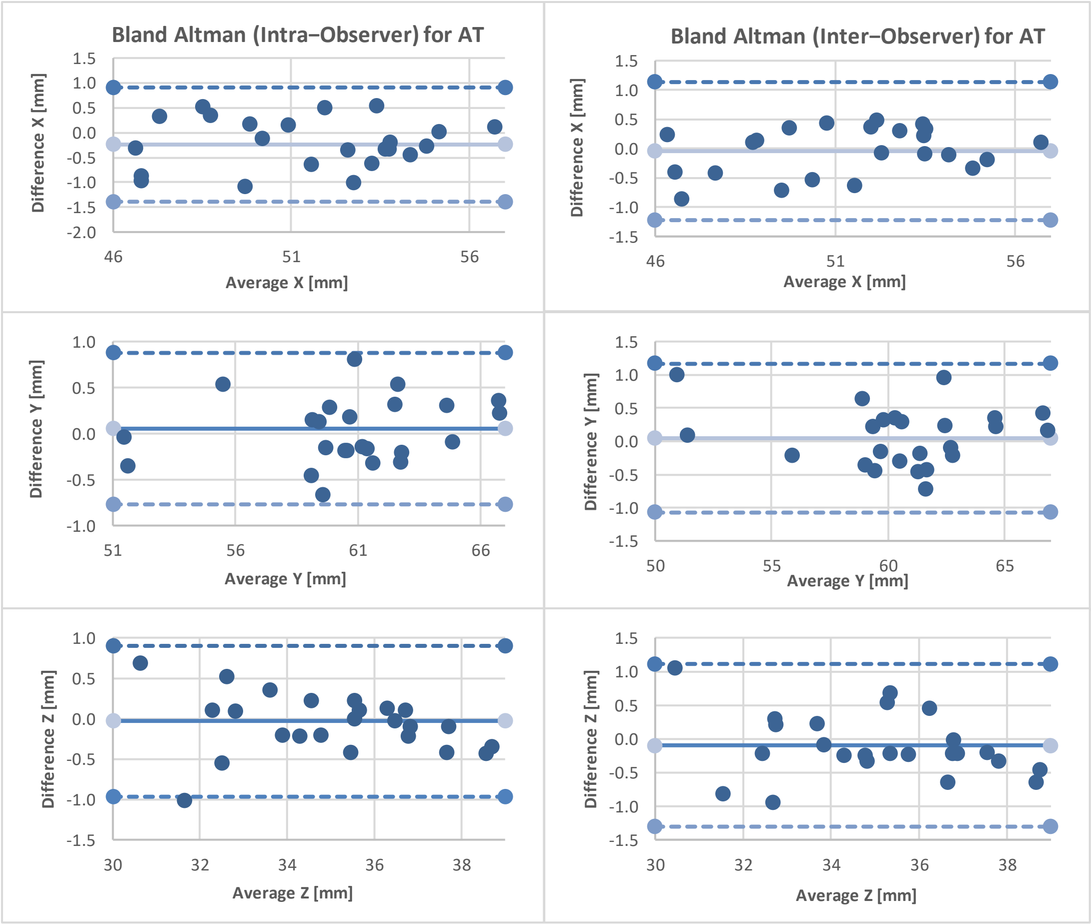

Bland Altman Plot for "Point IM"

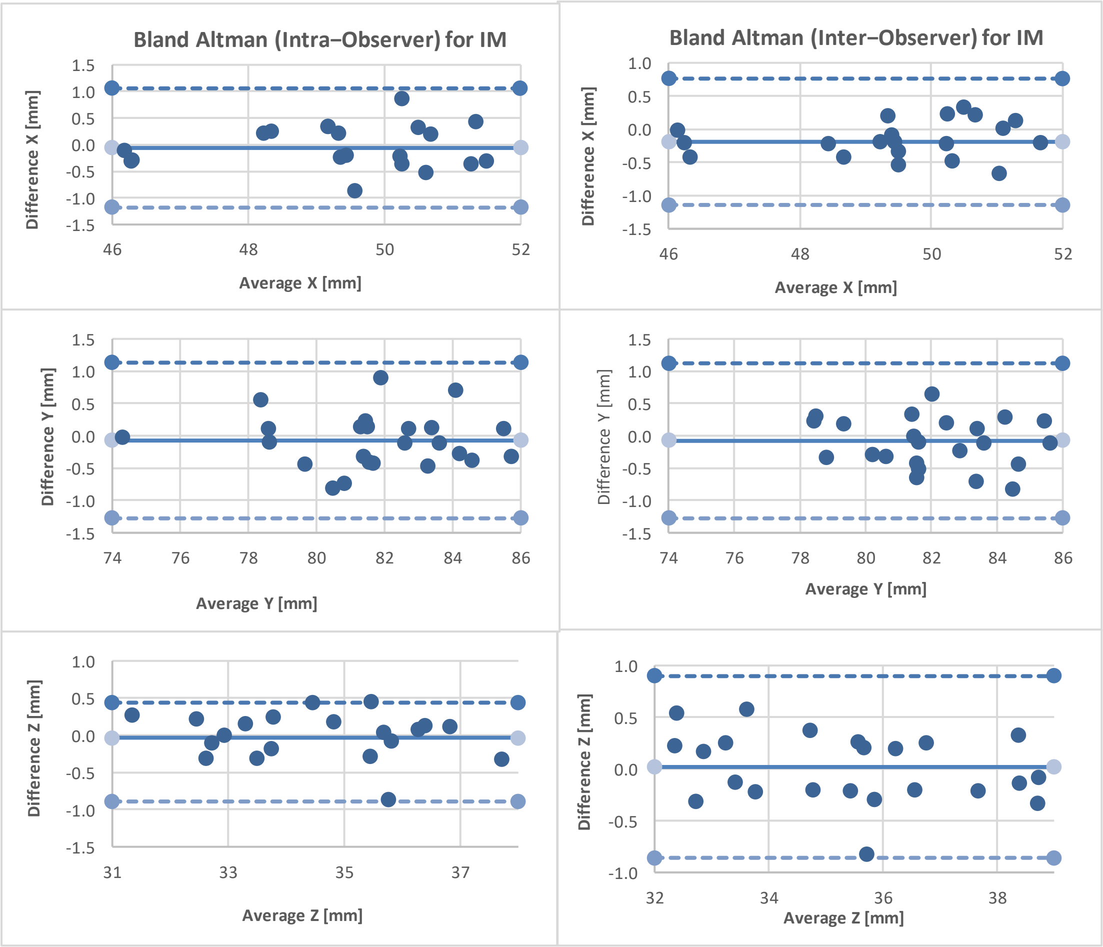

Bland Altman Plot for "Point AF"

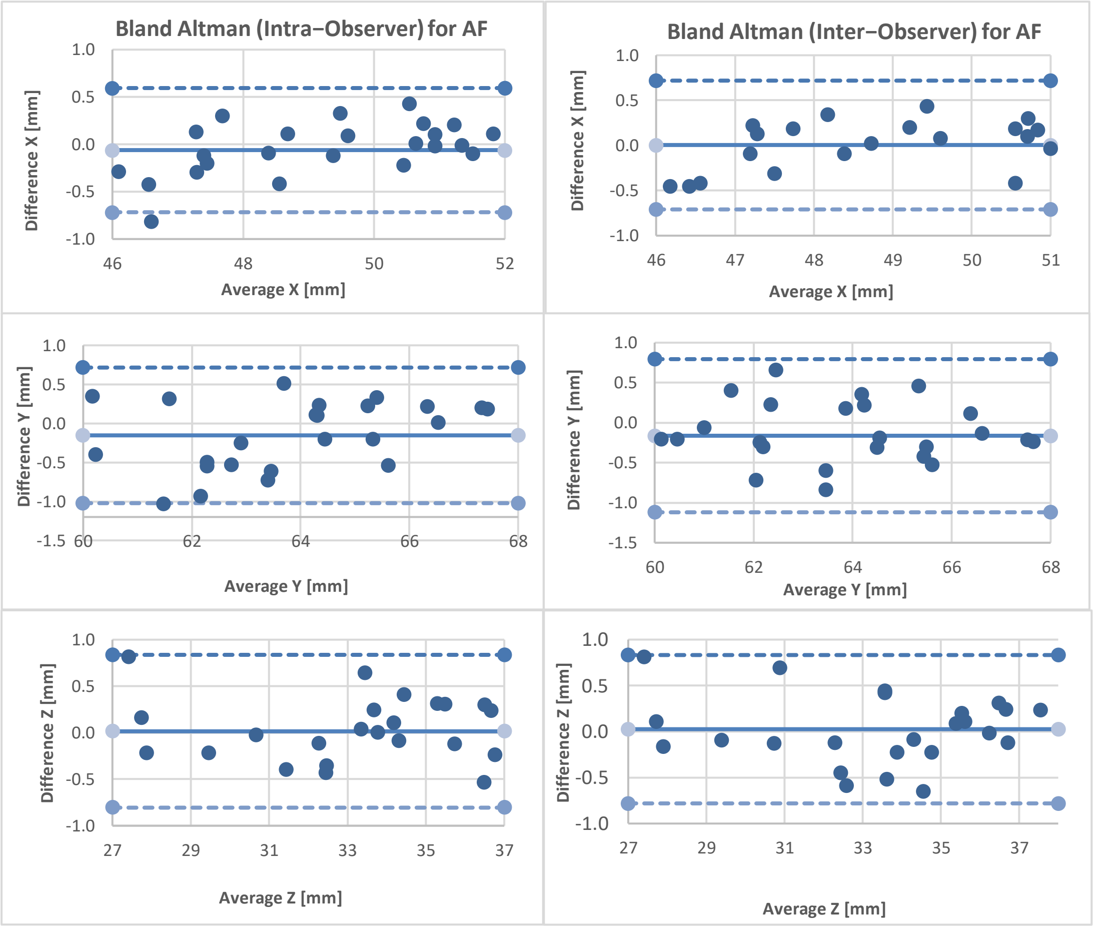

Bland Altman Plot for "Point PF"

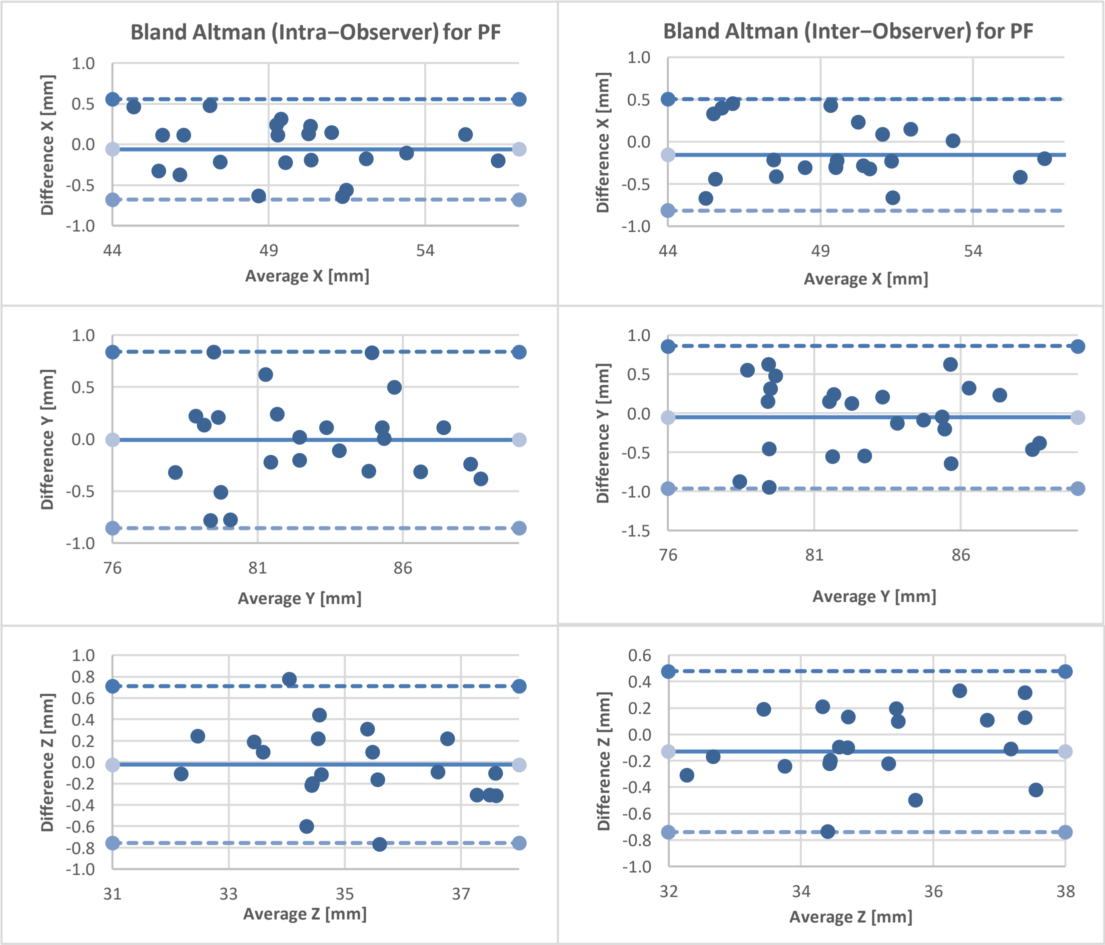

Bland Altman Plot for "Point AJSf"

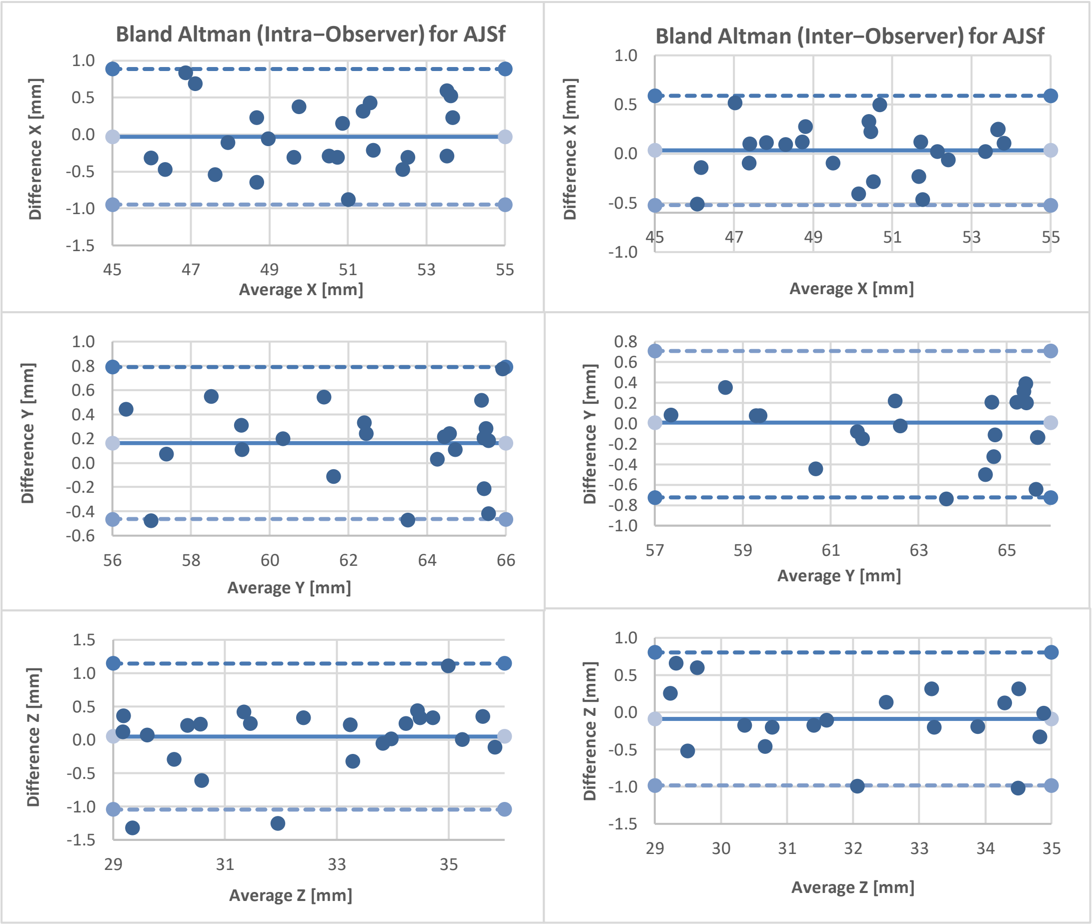

Bland Altman Plot for "Point AJSc"

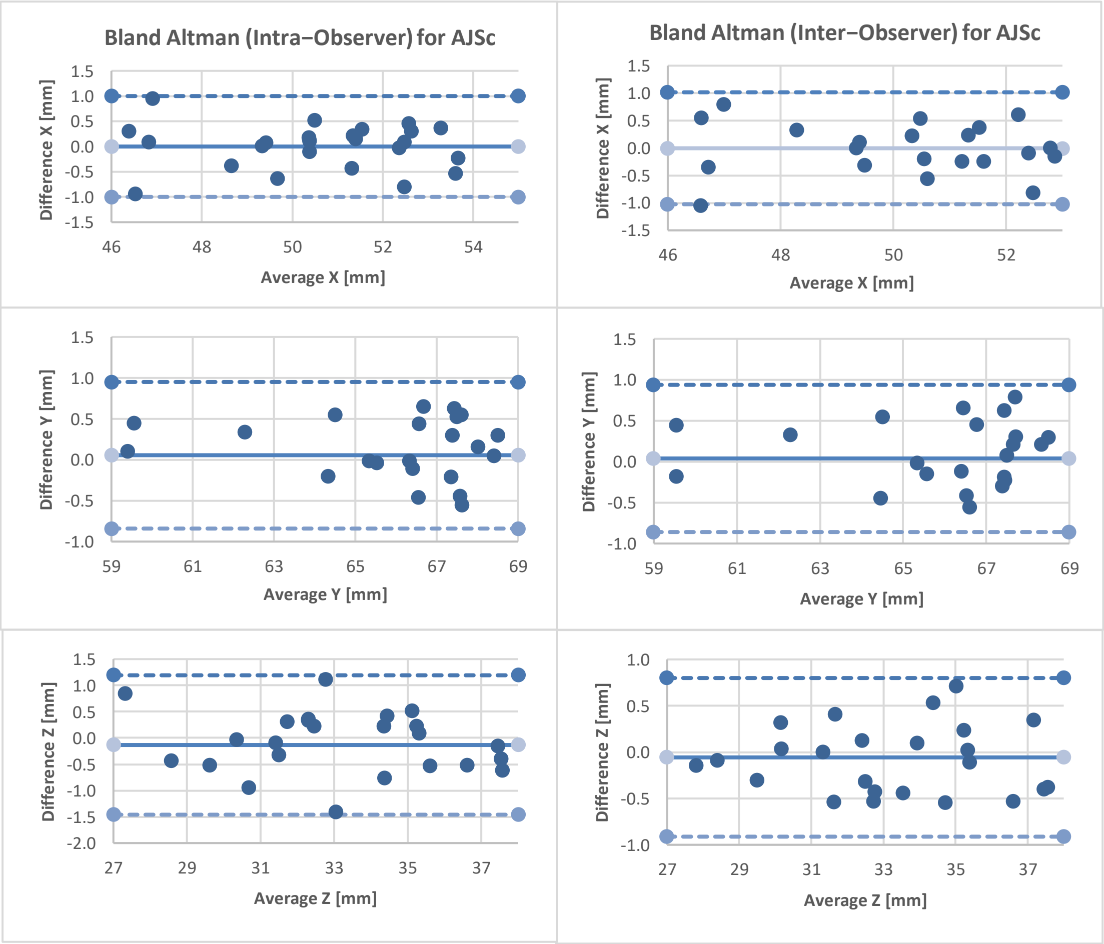

Bland Altman Plot for "Point PJSf"

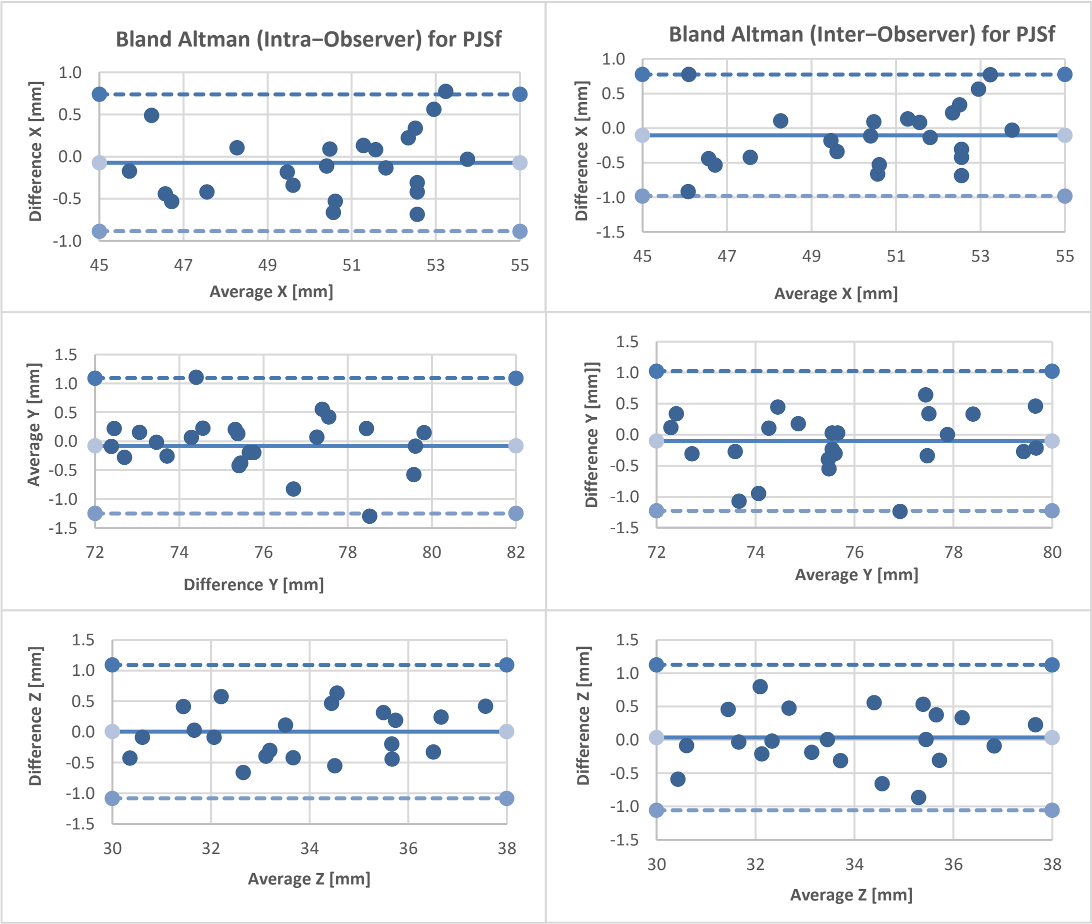

Bland Altman Plot for "Point PJSc"

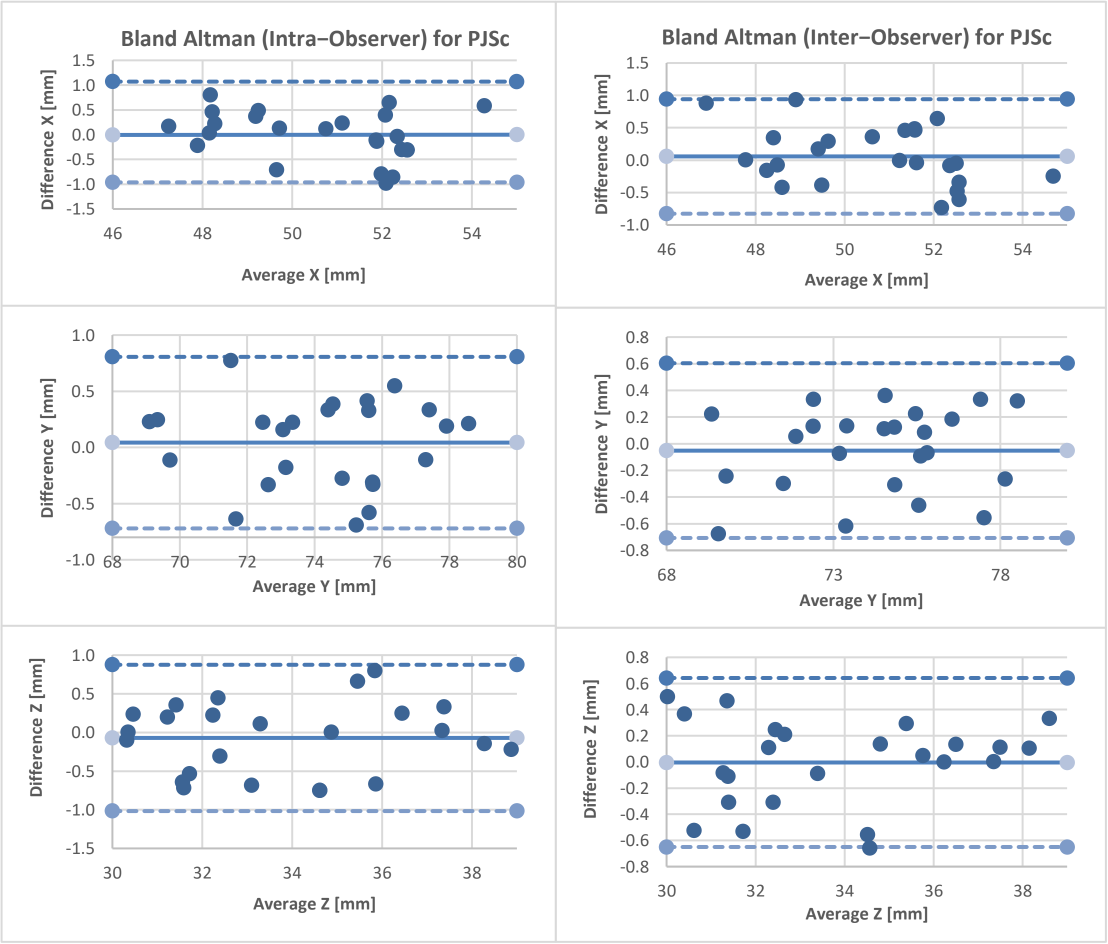

Supplement: Supplementary file 2 — Additional file 2. Bland-Altman plots in Intra and Inter-observer reliability for the TMJ landmarks. [file 12903_2022_2174_MOESM2_ESM.pdf]
